# Fabrication of Supercritical Antisolvent (SAS) Process-Assisted Fisetin-Encapsulated Poly (Vinyl Pyrrolidone) (PVP) Nanocomposites for Improved Anticancer Therapy

**DOI:** 10.3390/nano10020322

**Published:** 2020-02-13

**Authors:** Lin-Fei Chen, Pei-Yao Xu, Chao-Ping Fu, Ranjith Kumar Kankala, Ai-Zheng Chen, Shi-Bin Wang

**Affiliations:** 1 Department of Chemical Engineering and Pharmaceutical Engineering, College of Chemical Engineering, Huaqiao University, Xiamen 361021, China; 18013087024@stu.hqu.edu.cn (L.-F.C.); peiyaoxu@126.com (P.-Y.X.); ranjithkankala@hqu.edu.cn (R.K.K.); 2 Institute of Biomaterials and Tissue Engineering, Huaqiao University, Xiamen 361021, China; fuchp@hqu.edu.cn; 3 Fujian Provincial Key Laboratory of Biochemical Technology, Huaqiao University, Xiamen 361021, China

**Keywords:** supercritical fluid technology, fisetin, nanofabrication, antitumor

## Abstract

Due to its hydrophobicity, fisetin (FIS) often suffers from several limitations in terms of its applicability during the fabrication of pharmaceutical formulations. To overcome this intrinsic limitation of hydrophobicity, we demonstrate here the generation of poly (vinyl pyrrolidone) (PVP)-encapsulated FIS nanoparticles (FIS-PVP NPs) utilizing a supercritical antisolvent (SAS) method to enhance its aqueous solubility and substantial therapeutic effects. In this context, the effects of various processing and formulation parameters, including the solvent/antisolvent ratio, drug/polymer (FIS/PVP) mass ratio, and solution flow rate, on the eventual particle size as well as on distribution were investigated using a 2^3^ factorial experimental design. Notably, the FIS/PVP mass ratio significantly affected the morphological attributes of the resultant particles. Initially, the designed constructs were characterized systematically using various techniques (e.g., chemical functionalities were examined with Fourier-transform infrared (FTIR) spectroscopy, and physical states were examined with X-ray diffraction analysis (XRD) and differential scanning calorimetry (DSC) techniques). In addition, drug release as well as cytotoxicity evaluations in vitro indicated that the nanosized polymer-coated particles showed augmented performance efficiency compared to the free drug, which was attributable to the improvement in the dissolution rate of the FIS-PVP NPs due to their small size, facilitating a higher surface area over the raw form of FIS. Our findings show that the designed SAS process-assisted nanoconstructs with augmented bioavailability, have great potential for applications in pharmaceutics.

## 1. Introduction

Fisetin (FIS, C_15_H_10_O_6_), a widely spread flavonoid in many fruits and vegetables [1,2], has garnered enormous interest due to its numerous biological activities, including its antioxidant [3], antibacterial [4,5], anticancer, and neuroprotective effects [6], among other therapeutic properties. Specifically, in terms of antiproliferative effects, FIS is a highly active flavonoid against multiple types of cancers in various organs such as the colon, breasts, lungs, and liver [7,8,9,10,11,12]. As the most common and deadly cancer in women, breast cancer results in the deaths of numerous women worldwide. In this context, numerous research studies have recently demonstrated that FIS significantly induced breast cell growth inhibition, migration, and apoptosis by reversing epithelial-to-mesenchymal transition via the PTEN/Akt/GSK3 beta signal pathway [13], altering the PI3K/Akt pathway [14], and inhibiting matrix metalloproteinase expression [15]. Despite its substantial therapeutic effects, the limited solubility of FIS in aqueous media, which leads to bioavailability issues, has significantly hampered its applicability in the fabrication of pharmaceutical formulations [16,17].

In recent years, the fabrication of delivery systems at reduced size in the nanosized range has been of enormous interest in medicine, as this promises the improved bioavailability of insoluble drugs due to their enriched specific surface area of exposure to the surrounding physiological fluids [18,19]. The numerous conventional methods for the micronization or nanonization of drugs include spray-drying, mechanical crushing, and solvent evaporation, among others [20,21]. However, these techniques often suffer from notable limitations, including multi-step preparation, high-temperature operating conditions causing the degradation of thermosensitive compounds, and high amounts of residual solvent content that reduce the quality of the formulation, among others [20,21,22,23]. To this end, the supercritical fluid (SCF) technology has been increasingly recognized as a green alternative to various conventional techniques in nanoparticle formation and formulation design due to the utilization of green solvents such as water and carbon dioxide at their supercritical state, which results in no residual organic solvents in the end product, highly tunable processing conditions, and ease of operation (a single step), among other things [24,25,26,27,28,29]. Among the various SCFs available, supercritical carbon dioxide (SC-CO_2_) has been widely used in SCF-based micronization processes due to its mild critical conditions (*T*_c_ = 304.1 K, *P*_c_ = 7.38 MPa), the fact that it is environmentally friendly, and its low cost. Accordingly, SC-CO_2_ can be used as a solvent, cosolute, antisolvent, and dispersant in the fabrication of various drug delivery systems using drugs, genes, and proteins [30,31,32].

There are various SCF-assisted micronization techniques based on SC-CO_2_ behavior, and the supercritical antisolvent (SAS) process has been successfully used to prepare nanodosage forms of drugs (their polymeric composites in pharmaceutical formulations) [32,33,34]. In general, in the SAS process, solutes (drugs) are dissolved in organic solvents, and then the organic solvent and SC-CO_2_ diffuse into each other, leading to the occurrence of a high degree of supersaturation and the eventual precipitation of the solutes. In addition, the key factors affecting particle size as well as distribution and morphology include initial solution concentration, temperature, pressure, organic solvents, antisolvents, and the solution flow rate [26,35,36]. Due to these attributes, the SAS technique is considered to be an ideal tool for improving the solubility and bioavailability of insoluble drugs by conveniently regulating the operating conditions.

In the recent past, several formulations of FIS have been developed in an attempt to address its bioavailability issues, including liposomes, polymeric micelles, and dendrimers [1,37]. Along this line, the coprecipitation of water-insoluble drugs with hydrophilic polymers on a nanoscale can substantially improve the solubility of active drugs. Poly (vinyl pyrrolidone) (PVP) is one such hydrophilic synthetic polymer, and it is widely used as a carrier in the fabrication of controlled release systems to enhance the dissolution rate and solubility of poorly water-soluble drugs. Moreover, PVP is an excellent polymer (with the properties of biodegradability, biosafety, and biocompatibility) and has been promising in several pharmaceutical applications, such as wound dressing [38], tissue engineering [39], and drug delivery [33]. Because PVP retards crystal growth and stabilizes solids in a high-energy state, it is highly suitable as a biocompatible polymer in coprecipitating crystalline drugs [40]. In this framework, previous studies have reported the successful fabrication of PVP-based nanocomposites using the SAS process [33,41].

To improve the dissolution properties of FIS, micronized solid dispersions of FIS were fabricated using the SAS method in this work. Although the experimental conditions were optimized, the micronized FIS displayed irregular rod-like heterostructures. Since the morphology and particle size of the prepared FIS particles (using SAS) were not ideal enough, we developed a new formulation based on polymer-encapsulated FIS using the SAS system as a candidate. Herein we demonstrate the fabrication of FIS-PVP NPs using the SAS process to improve the bioavailability and anticancer efficacy of FIS. To optimize the formulation and processing parameters, we used a 2^3^ full-factor experimental design to investigate the effects of the solvent/antisolvent ratio (ethanol/dichloromethane (EtOH/DCM)), drug/polymer (FIS/PVP) mass ratio, and solution flow rate on the morphology of the nanoparticles. The potential performance efficiency of FIS was evaluated through an investigation of its dissolution behavior and antiproliferation activity in breast cancer cells in vitro.

## 2. Experimental Section

### 2.1. Materials

FIS (purity≥95%) was obtained from Dalian Meilun Biotechnology Co., Ltd. (Dalian, China). EtOH and DCM were purchased from Xilong Scientific Co., Ltd. (Shantou, China). Tween 80, potassium bromide (KBr), and PVP (molecular weight = 58,000) were obtained from Aladdin Co., Ltd. (Shanghai, China). Dulbecco’s modified eagle medium (DMEM) and trypsin-ethylenediaminetetraacetic acid (EDTA) were purchased from Biological Industries Co., Ltd. (Kibbutz Beit-Haemek, Israel). Methylthiazolyldiphenyl-tetrazolium bromide (MTT), phosphate-buffered saline (PBS), and an acridine orange/ethidium bromide (AO/EB) kit were obtained from Beijing Solarbio Science & Technology Co., Ltd. (Beijing, China). Human breast cancer (MDA-MB-231) cells were purchased from the Beina Chuanglian Biotechnology Institute (Beijing, China).

### 2.2. Fabrication of FIS-PVP NPs

**Figure 1:**
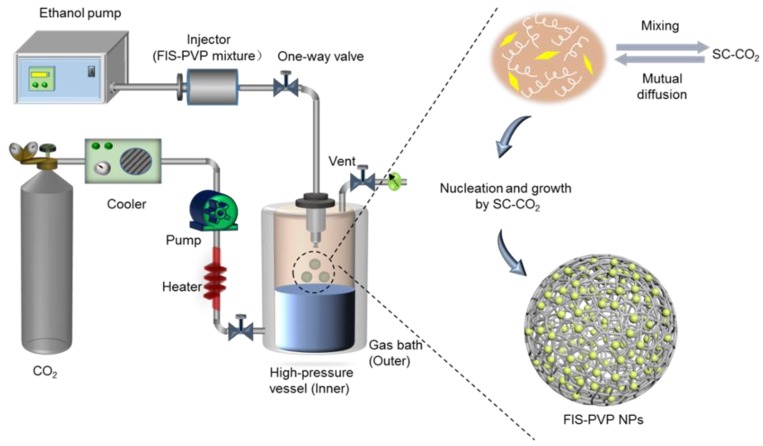
Schematic representation of the supercritical antisolvent (SAS) device set-up for the preparation of poly (vinyl pyrrolidone) (PVP)-encapsulated FIS nanoparticles (FIS-PVP NPs).

Figure 1 depicts an outline of the SAS apparatus set-up (SN3937782; Waters, Milford, MA, USA), and the processing is described briefly. Before entering the pump, CO_2_ was rapidly cooled to a liquid state through a condensation system, thus avoiding cavitation. Then, the liquefied CO_2_ was transported to a high-pressure vessel (HPV) by a high-pressure meter pump through an adjustment of the system parameters to the desired pressure and temperature for a supercritical state of CO_2_, which remained constant. The solution mixture was then pumped into the HPV through a specially designed nozzle. The final sample was collected through a stainless steel filter located at the bottom of the HPV.

In our work, the FIS-PVP NPs were prepared using the SAS process. Initially, FIS (20 mg) was dissolved in the EtOH/DCM solvent mixture (20 mL), and PVP was then added, resulting in a homogenous mixture solution of FIS/PVP with various mass ratios (1:2, 1:5, and 1:8). Then, the solution of FIS/PVP was sprayed into the HPV through a specially designed nozzle at a specific injection rate (0.5, 0.75, or 1 mL min^−1^). Finally, the fresh SC-CO_2_ was pumped for approximately 15 min to ensure the complete removal of the residual organic solvent. The HPV was then slowly depressurized, and the resultant FIS-PVP nanocomposites were collected.

During the SAS process, the effects of various critical variables on the particle morphology were determined, including the solvent/antisolvent ratio (EtOH/DCM), the FIS/PVP mass ratio, and the solution flow rate. To study the interactions of these three factors and their effects on the surface morphology as well as the particle size of the FIS-PVP nanocomposites, a 2^3^ factorial experiment was designed and an SAS-assisted fabrication was executed. The variance of the experimental data was analyzed by MINITAB software version 17 (Minitab Inc, Pittsburgh, PA, USA). By further optimizing the processing conditions, the experimental process for preparing particles with a fine morphology was determined.

### 2.3. Characterization of FIS-PVP NPs

Field-emission scanning electron microscopy (FE-SEM, S-4800, HITACHI, Tokyo, Japan) was used to observe the surface morphology and particle size of the FIS-PVP NPs at 5 kV and 10 mA. Before SEM observation, the sample was adhered to a conductive resin and sputtered with gold in a vacuum to obtain enough conductivity. The particle size and distribution of 500 particles in each sample were analyzed by Image Nano measurer software.

Fourier-transform infrared spectroscopy (FTIR, Nicolet iS50, Thermo Fisher Scientific, Waltham, MA, USA) was used to analyze the chemical functionalities of the drug/polymer composites after the SAS process. The scan wavenumber range was 4000–500 cm^−1^, and an average of 16 scan signals was considered to reduce the noise. The KBr pellet method was prepared by mixing powdered samples with the KBr matrix and compressing them with a hydraulic press.

Raw FIS, pure PVP, physical mixture of FIS+PVP, and FIS-PVP NPs were analyzed using an X-ray diffraction analyzer (XRD, SmartLa, Rigaku, Tokyo, Japan) to determine the crystallization behavior. The XRD curves were recorded in the 2*θ* range of 5–60° at a scanning speed of 10° min^−1^.

Differential scanning calorimetry (DSC, DSC 200F3, NETZSCH, Bavaria, Germany) was utilized to study the thermal behavior of raw FIS, pure PVP, and FIS-PVP NPs. Powdered samples of the designed composites (~4 mg) were accurately weighed, crimped into an aluminum pan, and subsequently heated from an ambient temperature to 400 °C at a rate of 10 °C/min.

### 2.4. In Vitro Dissolution Studies

The solubility measurements of raw FIS and FIS-PVP NPs were performed using the dialysis method. Briefly, accurately weighed samples containing an equal amount of FIS (2 mg) were placed into dialysis bags with a molecular weight cutoff (MWCO) point of 3.5 kDa. Then, the dialysis bags were dipped in 20 mL of PBS (0.5% Tween 80, pH 7.4) at 37 °C and placed in a rotary shaker spinning at 100 rpm. Further, aliquots of the dispersion medium (5 mL) were collected at predetermined intervals and instantaneously replenished with the same amount of fresh PBS. The amount of dissolved FIS was then determined by measuring the absorbance values at 364 nm using a UV–VIS spectrophotometer (UV-1800, Shimadzu, Kyoto, Japan). All release experiments were carried out in triplicate.

### 2.5. Antiproliferation Studies

MDA-MB-231 cells were cultivated in DMEM medium containing 10% (*v*/*v*) FBS, penicillin (100 units/mL), and streptomycin (100 μg/mL) and maintained in a humidified incubator (37 °C, 5% CO_2_). An MTT assay was used to investigate the effects of the FIS-PVP NPs and raw FIS at the same FIS concentration against the viability of the MDA-MB-231 cells. Briefly, the cells were first seeded into a 96-well plate (5 × 10^3^ cells per well) and incubated overnight. Further, the media in the wells were replaced with the fresh culture medium containing FIS-based nanocomposites at various concentrations and further incubated for another 24 h. Further, the MTT working solution (5 mg/mL, 20 μL) was added and incubated together for an additional 4 h. The formazan absorbance was then measured at 570 nm using a microplate reader (Multiskan EX; Thermo Fisher Scientific, Waltham, MA, USA), and the percentage viability of cells was then calculated based on the Equation (1):

(1) $$\mathrm{Cell}\mathrm{viability}(\%)=\;\frac{OD_{sample}}{OD_{control}}\times 100,$$

### 2.6. Apoptosis Analysis Using AO/EB Staining

The apoptosis of MDA-MB-231 cells was observed using fluorescence microscopy (NIKON, Ci-L, Tokyo, Japan) after staining with AO/EB to evaluate the antitumor effect. The cells (1 × 10^5^ cells per well) were seeded onto 24-well plates and allowed to adhere overnight. Then, the fresh medium containing raw FIS and FIS-PVP NPs at the same FIS concentration (40 μg/mL) was replaced for incubation with the cells for 24 h. After trypsin/EDTA treatment, live and dead cells were collected and incubated with AO/EB mixed solution in the dark for 15 min at room temperature. Finally, the images were captured for further analysis.

### 2.7. Statistical Analysis

All experimental results are expressed as mean ± standard deviation (*n* = 3) values and were analyzed using a one-way analysis of variance (ANOVA) after a Tukey test (*p* < 0.05) (using GraphPad Prism (Version 7.0, GraphPad Software, San Diego, CA, USA)).

## 3. Results and Discussion

### 3.1. Effect of PVP on the Morphology of FIS Particles

**Figure 2:**
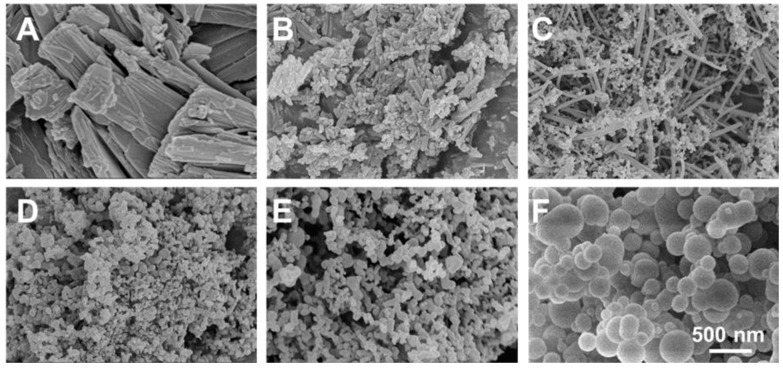
SEM photographs of (**A**) raw FIS, (**B**) SAS process-assisted FIS, and FIS-PVP particles at different FIS/PVP mass ratios (*w*/*w*) of (**C**) 1:0.5, (**D**) 1:1, (**E**) 1:2, and (**F**) 1:5.

Prior to the optimization of the various parameters, a preliminary investigation was performed by regulating one of the key parameters—the mass ratio of the substrates, FIS and PVP—to evaluate the feasibility of FIS encapsulation in PVP using the SAS process (Table 1). Notably, all SAS experiments were carried out using critical conditions, i.e., a pressure of 100 bar, a temperature of 45 °C, and a CO_2_ flow rate of 35 g/min. As is shown in Figure 2, the corresponding SEM images of raw FIS and other FIS-based nanocomposites under different operating conditions showed large crystals with an irregular shape (in the case of raw FIS) (Figure 2A), while the SAS process-assisted FIS particles displayed irregular rod-like structures (Figure 2B). Compared to raw FIS, the SAS process significantly reduced the particle size, and the processing was required to improve solubility. To this end, the PVP-encapsulated FIS in similar experimental conditions resulted in different morphologies of crystals and irregular particles, with a FIS/PVP ratio of 1:0.5 *w/w*, which could have been due to the FIS and PVP constructs themselves, due to their nature. These consequences indicated that the coprecipitation was unsuccessful, since the two compounds were precipitated separately (Figure 2C). Further, with changes to the FIS/PVP ratio (to 1:1 *w*/*w*), particles with reduced sizes in a spherical shape only were obtained (Figure 2D). With further increases in the PVP concentration, particles (appropriately) in a spherical shape were obtained; however, the particle size was amplified significantly (Figure 2E,F). These experimental results showed that, despite the poor candidature of FIS for micronization, the addition of a certain amount of PVP resulted in uniformly sized, discrete, and spherically shaped nanocomposites, due to the precipitation of amorphous PVP over FIS.

### 3.2. Experimental Optimization

**Figure 3:**
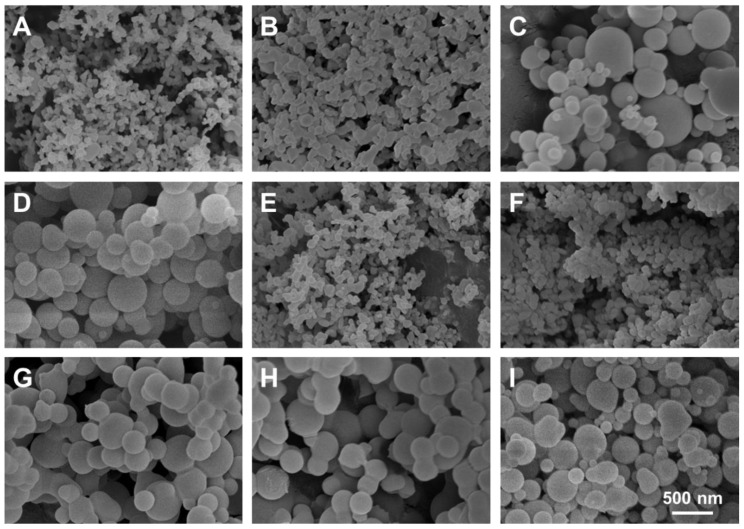
SEM images of samples prepared under different operating conditions (as shown in Table 3). (**A**) 1:2, 1:2, 0.5 mL min^−1^; (**B**) 2:1, 1:2, 0.5 mL min^−1^; (**C**) 1:2, 1:8, 0.5 mL min^−1^; (**D**) 2:1, 1:8, 0.5 mL min^−1^; (**E**) 1:2, 1:2, 1 mL min^−1^; (**F**) 2:1, 1:2, 1 mL min^−1^; (**G**) 1:2, 1:8, 1 mL min^−1^; (**H**) 2:1, 1:8, 1 mL min^−1^; and (**I**) 1:1, 1:5, 0.75 mL min^−1^ (the operating parameters are EtOH/DCM (*v/v*), FIS/PVP (*w/w*), and the flow rate of the solution mixture (mL min^−1^), respectively).

**Figure 4:**
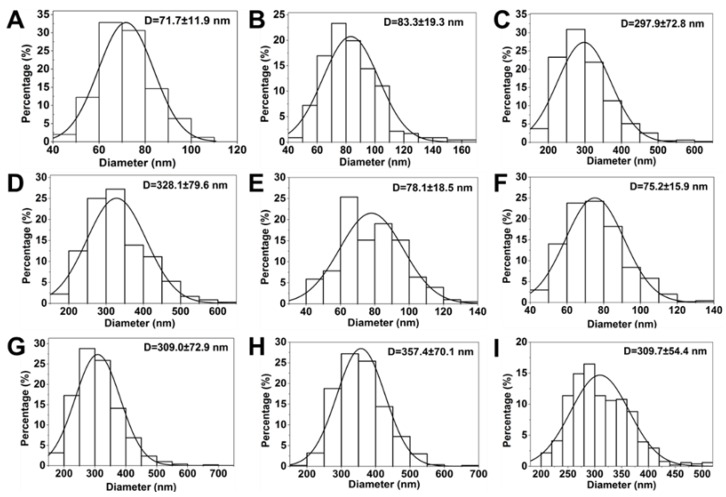
Histograms showing the particle size distribution of samples prepared under different operating conditions (as shown in Table 3). (**A**) 1:2, 1:2, 0.5 mL min^−1^; (**B**) 2:1, 1:2, 0.5 mL min^−1^; (**C**) 1:2, 1:8, 0.5 mL min^−1^; (**D**) 2:1, 1:8, 0.5 mL min^−1^; (**E**) 1:2, 1:2, 1 mL min^−1^; (**F**) 2:1, 1:2, 1 mL min^−1^; (**G**) 1:2, 1:8, 1 mL min^−1^; (**H**) 2:1, 1:8, 1 mL min^−1^; and (**I**) 1:1, 1:5, 0.75 mL min^−1^ (the operating parameters are EtOH/DCM (*v/v*), FIS/PVP (*w/w*), and the flow rate of the solution mixture (mL min^−1^), respectively).

A 2^3^ full factorial experimental design was considered based on the levels and codes of the Minitab analysis design mentioned in Table 2. Changes in the FIS-PVP NPs’ mean size and span at different run orders based on the Minitab design are displayed in Table 3. Figure 3 depicts SEM images of the samples prepared under the different operating conditions specified in Table 3. Figure 4 shows the particle size distribution of FIS-PVP NPs, which corresponds to the images in Figure 3. FIS-PVP nanocomposites of various shapes and sizes were prepared by regulating the key parameters. According to Table 3 and Figure 3, it was more intuitive to prove that the SAS operation conditions had significant effects on particle morphology and particle size distribution. After SAS treatment, FIS and PVP precipitated into nanosized composites, in which the drug/polymer mass ratio was the dominant factor affecting the size of the FIS-PVP NPs. However, the influence of the EtOH/DCM ratio and the solution flow rate on the particle morphology was partial. In the preliminary experiments (Figure 2), we observed that FIS alone did not result in a nanometer size range using the SAS process; however, with an increase in the proportion of PVP in the mixed system of FIS and PVP (FIS/PVP mass ratio from 1:0 to 1:1), the obtained samples gradually tended to be spherical, which could have been due to the presence of PVP in the solution mixture that promoted the precipitation of FIS particles in an amorphous way. When the concentration of PVP in the system was enough to inhibit the crystallization of the drugs, this resulted in a highly supersaturated state, and the drugs precipitated into small-sized particles. Moreover, our findings were in accordance with the reported literature that the polymer PVP can interfere with the crystallization kinetics of some drugs, resulting in amorphous composites [40,42].

Furthermore, the FIS/PVP mass ratios were adjusted to 1:2 and 1:8, while the operating conditions, including the EtOH/DCM ratio and solution flow rate, were unchanged. SEM images of the obtained samples showed that, with the continuous increase in the PVP concentration (mass ratio 1:2), the SAS process resulted in smaller-sized, spherically shaped FIS-PVP nanocomposites (Figure 3A). Moreover, at a FIS/PVP mass ratio of 1:8, the resultant nanocomposites were spherical and discrete, with a uniform size distribution (Figure 3C). In addition, the particle size distribution and average particle size were substantially increased. Clearly, with the higher proportion of PVP in the mixture, the particle size of the FIS-PVP NPs increased significantly, which could be attributable to the increase in the viscosity of the mixture due to excess PVP. Such an enhanced amount of PVP would also result in the slowing down of the nucleation rate, eventually leading to the formation of agglomerates [43].

**Figure 5:**
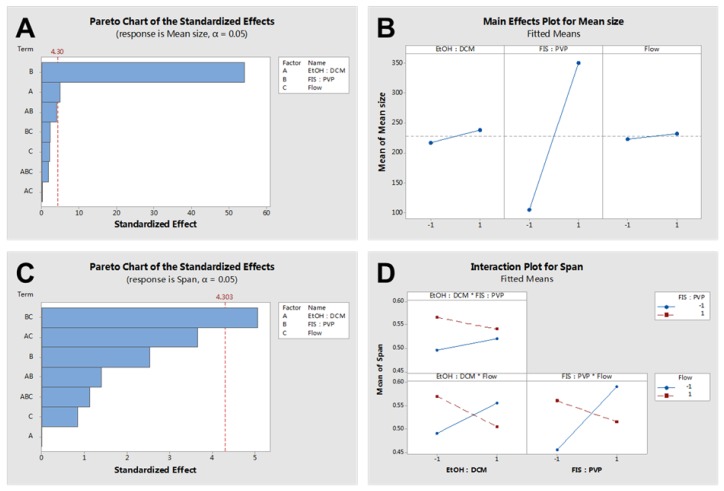
(**A**) Standardized effects of the factors on mean size, (**B**) a main effects plot for mean size, (**C**) standardized effects of the factors on span, and (**D**) an interaction plot for the span.

Figure 5 shows the influence of various factors on the morphology of the obtained FIS-PVP NPs (using statistical analysis). As is depicted in Figure 5A, factors A (solvent/antisolvent) and B (FIS/PVP mass ratio) had a significant effect on the mean size of the FIS-PVP NPs. It could be observed in the experimental results (Figure 5B) that the average particle size of the FIS-PVP NPs increased with an increase in the FIS/PVP mass ratio and the volume ratio of the solvent/antisolvent. Figure 5C shows that those factors had no significant effect on the span: the most significant effect was the interaction between factor B and C (Figure 5D). Moreover, the FIS-PVP NPs displayed a homogeneous morphology, while factors B and C were both at a low level at the same time.

Based on an analysis of the SEM observations, we believe that the FIS/PVP mass ratio was the main factor, playing a crucial role in significantly affecting the particle morphology. By controlling the EtOH/DCM ratio and solution flow rate and by adjusting the drug/polymer ratio, the SAS process at the optimized experimental conditions resulted in PVP-encapsulated FIS nanocomposites with excellent morphology. Therefore, it should be noted that the formation of FIS-PVP NPs with appropriate morphological attributes for increasing the solubility and subsequent bioavailability of drugs could be attained by regulating the FIS/PVP mass ratio in the SAS process. Accordingly, optimal formulation and processing parameters (EtOH/DCM of 1:2, a FIS/PVP mass ratio of 1:2, and a solution flow rate of 0.5 mL min^−1^) were selected to carry out the subsequent experiments. Moreover, the FIS loading efficiency in the obtained nanocomposite formulation under the optimal critical conditions of the SAS process was enumerated as 42%.

### 3.3. Characterizations

**Figure 6:**
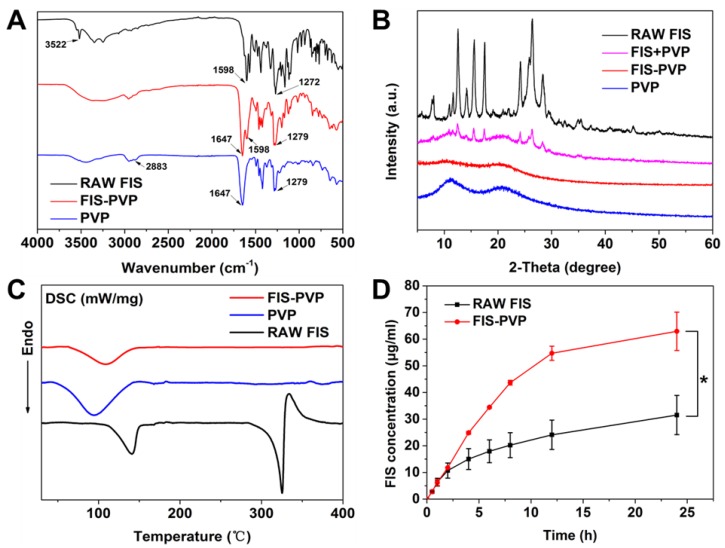
(**A**) FTIR spectra of raw FIS, pure PVP, and FIS-PVP NPs. (**B**) XRD patterns of raw FIS, pure PVP, the physical mixture of FIS+PVP, and the SAS-processed FIS-PVP NPs. (**C**) DSC thermograms of raw FIS, pure PVP, and the SAS process-based FIS-PVP NPs. (**D**) Amount of soluble FIS in raw FIS as well as that released from the SAS process-assisted FIS-PVP NPs.

The FIS-PVP nanocomposite formulation obtained at the optimal parameters were then systematically characterized using various techniques. FTIR spectra of raw FIS, pure PVP, and FIS-PVP NPs were recorded to analyze the chemical functionalities and the influence of the SCF process (Figure 6A). Absorption bands at 3522, 1598, and 1272 cm^−1^ could be ascribed to O-H stretching, C=C stretching, and C-O-H bending vibrations, respectively, indicating the characteristic functional groups of FIS. On the other hand, the chemical functionalities of the C-H, C=O, and C-N vibrations representing PVP could be observed at 2883, 1647, and 1279 cm^−1^. However, some of these characteristic peaks disappeared in the spectrum of FIS-PVP NPs, which might have been due to overlapping of the spectra. Further, the crystallization behavior of raw FIS, pure PVP, the physical mixture of PVP+FIS, and the FIS-PVP NPs were analyzed using XRD patterns (Figure 6B). It was observed from the XRD patterns that the untreated FIS had a high-intensity characteristic diffraction peak, indicating its crystalline nature, while the characteristic peak strength of FIS was weakened due to the presence of amorphous PVP in the physical mixture [40]. However, after the SAS processing, this crystalline FIS completely disappeared, indicating that the FIS existed amorphously in the precipitated samples [44]. It could be concluded that the nature of FIS could be changed from crystalline to amorphous during the SAS process. In addition, the DSC thermograms of raw FIS, pure PVP, and the FIS-PVP NPs are shown in Figure 6C. The thermal spectrum of raw FIS showed a sharp heat absorption peak at 330 °C, which could be ascribed to the melting point of crystalline FIS, while the pure PVP exhibited a wide endothermic peak at 50 to 100 °C due to the process of water molecular loss in the test sample. Moreover, a thermal analysis atlas of the SAS process-assisted FIS-PVP nanoconjugate group resulted in a gentle melting point range of FIS with no significant heat absorption peak, indicating the amorphous nature of the composites after the SAS process. These DSC experimental results were in agreement with the XRD patterns, and the nature of the eventual formulation could be conducive for drug delivery applications.

### 3.4. Solubility and Dissolution Rate Investigations

To confirm whether the solubility of FIS was significantly improved after SAS, the release of FIS from the SAS process-assisted FIS-PVP nanoconjugates was investigated by suspending the corresponding samples in PBS (pH 7.4) and comparing them to the solubility and dissolution rate of raw FIS (equivalent to the loading amount of FIS in the FIS-PVP composites) in similar conditions. As shown in Figure 6D, the dissolution rate of raw FIS was relatively slow, approximately 31.5 μg/mL in 24 h. In contrast, the FIS-PVP NP group showed a distinct dissolution behavior, with a higher release of FIS around 63 μg/mL, which was approximately two-fold higher than that of raw FIS in the same exposure time. These consequences of improved solubility and an improved FIS dissolution rate could be ascribed to the formation of smaller-sized FIS-PVP NPs with an increased specific surface area of exposure and to the transformation of the physical state from crystalline to amorphous, which in turn significantly affected the bioavailability [36]. On the one hand, the presence of a water-soluble amorphous polymer stabilizing the solid in a high-energy state might force the FIS to be micronized into smaller particles through the SAS process, thus enlarging the surface area of the FIS. On the other hand, the transformation of the state of the drug from crystalline to amorphous, a process of increasing entropy, leads to thermodynamic instability, which might also be a reason for the dissolution of the FIS [45].

### 3.5. Antiproliferation Studies

**Figure 7:**
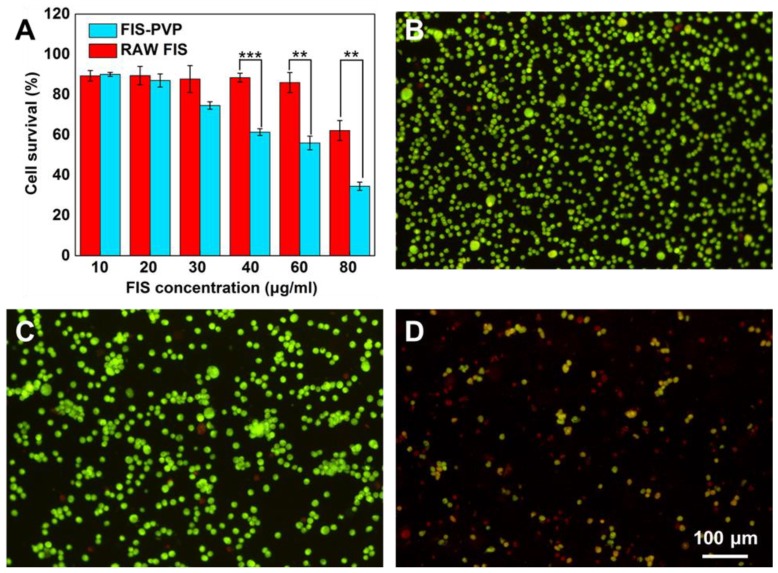
In vitro cytotoxicity study of FIS-PVP NPs. (**A**) Relative viability of MDA-MB-231 after treatment with FIS-PVP NPs and raw FIS. Fluorescence images of MDA-MB-231 cell staining with AO/EB after various treatments (the live cells are green, and the dead cells are red). (**B**) Negative control (media), (**C**) raw FIS, and (**D**) the FIS-PVP NPs.

Numerous studies have shown that FIS possesses certain anticancer effects. Moreover, PVP, a biocompatible polymer, has been widely used in drug delivery systems to fabricate various anticancer formulations [41,46,47]. To explore these aspects, the cytotoxicity of FIS-PVP NPs and raw FIS against MDA-MB-231 cells was assessed. After 24 h of incubation, raw FIS and FIS-PVP NPs showed dose-dependent growth inhibition (Figure 7A). It is worth noting that at the same FIS concentration of 80 μg/mL, the cell survival rate of the raw FIS treatment group was still as high as 62%, while that of the FIS-PVP nanoconjugate treatment group was only 35%, demonstrating that the FIS-PVP NPs obtained through the SAS process showed a more obvious inhibitory effect on cancer cells than did raw FIS. Compared to raw FIS, the improved anticancer effect of FIS-PVP NPs within 24 h was mutually corroborated by the solubility and dissolution rate results: the FIS-PVP NP group displayed an FIS concentration higher (by approximately two-fold) than that of the raw FIS at 24 h. Studies have shown that FIS can promote cancer cell apoptosis by affecting a variety of signaling pathways, which explains our experimental results [8,48]. In addition, similar cell survival results were observed under a fluorescence microscope after adding the AO/EB dual stain (Figure 7B–D). It was evident from the experimental results that a significant number of apoptotic and dead cells in the FIS-PVP NP group were observed (apoptotic cells in yellow and dead cells in red). The improved cytotoxicity of the FIS-PVP NPs was mutually corroborated by the aforementioned experimental results: the increased solubility and dissolution rate of the drugs substantially improved their bioavailability, with anticancer effects better than those of the raw drug molecules.

## 4. Conclusions

In this study, we successfully produced FIS-PVP NPs using the SAS process. The results of the 2^3^ full-factor experimental design showed that the drug/polymer (FIS/PVP) mass ratio was the dominant factor affecting particle morphology. Moreover, the presence of PVP promoted the transition of FIS from a crystalline to an amorphous state, and the resultant nanoconjugates were contained in smaller-sized particles through regulation of the amount of PVP. Dissolution tests confirmed a significant improvement in the solubility of FIS after SAS, which was roughly twice that of raw FIS. Finally, the FIS-PVP NPs showed potential antiproliferative efficacy in breast cancer cells. In conclusion, the SAS process may provide an effective way forward for the development of innovative FIS-based nanoformulations.

## Figures and Tables

**Table 1:** Influence of experimental conditions (FIS/PVP mass ratio) on the morphology of the resultant products at a solution flow rate of 1 mL min^−1^.

Parameter	1	2	3	4	5	6
FIS/PVP Mass ratio (*w*/*w*)	raw FIS	1:0	1:0.5	1:1	1:2	1:5
Morphology	strip shape	rod-like	irregular	irregular	spherical	spherical

**Table 2:** Experimental factors and levels of the Minitab analysis design. DCM: dichloromethane.

Level	Code	A Solvent/Antisolvent (EtOH/DCM) (*v*/*v*)	B FIS/PVP Mass Ratio (*w*/*w*)	C Solution Flow Rate (mL min^−1^)
High	+1	2:1	1:8	1
Medium	0	1:1	1:5	0.75
Low	−1	1:2	1:2	0.5

**Table 3:** Experimental results based on a Minitab full factorial design.

Run Order	Blocks	A	B	C	Mean Size of FIS-PVP NPs (nm)	Span (D90–D10)/D50
1	1	−1	−1	−1	71.7 ± 11.9	0.43
2	1	1	-1	−1	83.3 ± 19.3	0.54
3	1	−1	1	−1	297.9 ± 72.87	0.61
4	1	1	1	−1	328.1 ± 79.6	0.63
5	1	−1	−1	1	78.1 ± 18.5	0.62
6	1	1	−1	1	75.2 ± 15.9	0.56
7	1	−1	1	1	309.0 ± 72.9	0.58
8	1	1	1	1	357.4 ± 70.1	0.51
9	1	0	0	0	309.7 ± 54.4	0.43
10	1	0	0	0	300.0 ± 56.9	0.45
11	1	0	0	0	297.6 ± 53.7	0.48

Note: A—solvent/antisolvent (EtOH/DCM, *v*/*v*), B—FIS/PVP mass ratio (*w*/*w*), and C—solution flow rate (ml min^−1^) following the corresponding levels represented in Table 2.

## References

[1] Mehta P., Pawar A., Mahadik K., Bothiraja C. (2018). Emerging novel drug delivery strategies for bioactive flavonol fisetin in biomedicine. Biomed. Pharmacother..

[2] Kashyap D., Sharma A., Sak K., Tuli H.S., Buttar H.S., Bishayee A. (2018). Fisetin: A bioactive phytochemical with potential for cancer prevention and pharmacotherapy. Life Sci..

[3] Khan N., Syed D.N., Ahmad N., Mukhtar H. (2012). Fisetin: A dietary antioxidant for health promotion. Antioxid. Redox Signal..

[4] Gabor M., Eperjessy E. (1966). Antibacterial effect of fisetin and fisetinidin. Nature.

[5] Wang J.F., Qiu J.Z., Tan W., Zhang Y., Wang H.S., Zhou X., Liu S., Feng H.H., Li W.H., Niu X.D. (2015). Fisetin inhibits listeria monocytogenes virulence by interfering with the oligomerization of listeriolysin o. J. Infect. Dis..

[6] Chiruta C., Schubert D., Dargusch R., Maher P. (2012). Chemical modification of the multitarget neuroprotective compound fisetin. J. Med. Chem..

[7] Khan N., Mukhtar H. (2015). Dietary agents for prevention and treatment of lung cancer. Cancer Lett..

[8] Chen Y., Wu Q., Song L., He T., Li Y., Li L., Su W., Liu L., Qian Z., Gong C. (2015). Polymeric micelles encapsulating fisetin improve the therapeutic effect in colon cancer. ACS Appl. Mater. Interfaces.

[9] Pawar A., Singh S., Rajalakshmi S., Shaikh K., Bothiraja C. (2018). Development of fisetin-loaded folate functionalized pluronic micelles for breast cancer targeting. Artif. Cells Nanomed. Biotechnol..

[10] Feng C., Yuan X., Chu K., Zhang H., Ji W., Rui M. (2019). Preparation and optimization of poly (lactic acid) nanoparticles loaded with fisetin to improve anti-cancer therapy. Int. J. Biol. Macromol..

[11] Kang K.A., Piao M.J., Hewage S., Ryu Y.S., Oh M.C., Kwon T.K., Chae S., Hyun J.W. (2016). Fisetin induces apoptosis and endoplasmic reticulum stress in human non-small cell lung cancer through inhibition of the mapk signaling pathway. Tumor Biol..

[12] Liu Y.S., Chang Y.C., Kuo W.W., Chen M.C., Hsu H.H., Tu C.C., Yeh Y.L., Viswanadha V.P., Liao P.H., Huang C.Y. (2019). Inhibition of protein phosphatase 1 stimulates noncanonical er stress eif2 alpha activation to enhance fisetin-induced chemosensitivity in hdac inhibitor-resistant hepatocellular carcinoma cells. Cancers.

[13] Li J., Gong X., Jiang R., Lin D., Zhou T., Zhang A.J., Li H.Z., Zhang X., Wan J.Y., Kuang G. (2018). Fisetin inhibited growth and metastasis of triple-negative breast cancer by reversing epithelial-to-mesenchymal transition via pten/akt/gsk3 beta signal pathway. Front. Pharmacol..

[14] Guo G., Zhang W., Dang M., Yan M., Chen Z. (2018). Fisetin induces apoptosis in breast cancer mda-mb-453 cells through degradation of her2/neu and via the pi3k/akt pathway: Guo et al. J. Biochem. Mol. Toxicol..

[15] Tsai C.F., Chen J.H., Chang C.N., Lu D.Y., Chang P.C., Wang S.L., Yeh W.L. (2018). Fisetin inhibits cell migration via inducing ho-1 and reducing mmps expression in breast cancer cell lines. Food Chem. Toxicol..

[16] Seguin J., Brulle L., Boyer R., Lu Y.M., Romano M.R., Touil Y.S., Scherman D., Bessodes M., Mignet N., Chabot G.G. (2013). Liposomal encapsulation of the natural flavonoid fisetin improves bioavailability and antitumor efficacy. Int. J. Pharm..

[17] Kadari A., Gudem S., Kulhari H., Bhandi M.M., Borkar R.M., Kolapalli V.R.M., Sistla R. (2017). Enhanced oral bioavailability and anticancer efficacy of fisetin by encapsulating as inclusion complex with hp beta cd in polymeric nanoparticles. Drug Deliv..

[18] Liu C.-G., Han Y.-H., Zhang J.-T., Kankala R.K., Wang S.-B., Chen A.-Z. (2019). Rerouting engineered metal-dependent shapes of mesoporous silica nanocontainers to biodegradable janus-type (sphero-ellipsoid) nanoreactors for chemodynamic therapy. Chem. Eng. J..

[19] Kankala R.K., Liu C.-G., Yang D.-Y., Wang S.-B., Chen A.-Z. (2020). Ultrasmall platinum nanoparticles enable deep tumor penetration and synergistic therapeutic abilities through free radical species-assisted catalysis to combat cancer multidrug resistance. Chem. Eng. J..

[20] Rasenack N., Muller B.W. (2004). Micron-size drug particles: Common and novel micronization techniques. Pharm. Dev. Technol..

[21] Vogt M., Kunath K., Dressman J.B. (2008). Dissolution enhancement of fenofibrate by micronization, cogrinding and spray-drying: Comparison with commercial preparations. Eur. J. Pharm. Biopharm..

[22] Kim J.-S., Kim M.-S., Park H.J., Jin S.-J., Lee S., Hwang S.-J. (2008). Physicochemical properties and oral bioavailability of amorphous atorvastatin hemi-calcium using spray-drying and sas process. Int. J. Pharm..

[23] Chen B.Q., Kankala R.K., Zhang Y., Xiang S.T., Tang H.X., Wang Q., Yang D.Y., Wang S.B., Zhang Y.S., Liu G. (2020). Gambogic Acid Augments Black Phosphorus Quantum Dots (BPQDs)-Based Synergistic Chemo-Photothermal Therapy through Downregulating Heat Shock Protein Expression. Chem. Eng. J..

[24] Kankala R.K., Chen B.Q., Liu C.G., Tang H.X., Wang S.B., Chen A.Z. (2018). Solution-enhanced dispersion by supercritical fluids: An ecofriendly nanonization approach for processing biomaterials and pharmaceutical compounds. Int. J. Nanomed..

[25] Chen B.Q., Kankala R.K., Chen A.Z., Yang D.Z., Cheng X.X., Jiang N.N., Zhu K., Wang S.B. (2017). Investigation of silk fibroin nanoparticle-decorated poly(l-lactic acid) composite scaffolds for osteoblast growth and differentiation. Int. J. Nanomed..

[26] Kankala R.K., Zhang Y.S., Wang S.-B., Lee C.-H., Chen A.-Z. (2017). Supercritical fluid technology: An emphasis on drug delivery and related biomedical applications. Adv. Healthc. Mater..

[27] Chen B.-Q., Kankala R.K., Wang S.-B., Chen A.-Z. (2018). Continuous nanonization of lonidamine by modified-rapid expansion of supercritical solution process. J. Supercrit. Fluids.

[28] Pessi J., Lassila I., Meriläinen A., Räikkönen H., Hæggström E., Yliruusi J. (2016). Controlled expansion of supercritical solution: A robust method to produce pure drug nanoparticles with narrow size-distribution. J. Pharm. Sci..

[29] Lane M.K.M., Zimmerman J.B. (2019). Controlling metal oxide nanoparticle size and shape with supercritical fluid synthesis. Green Chem..

[30] Lin X.F., Kankala R.K., Tang N., Xu P.Y., Hao L.Z., Yang D.Y., Wang S.B., Zhang Y.S., Chen A.Z. (2019). Supercritical fluid-assisted porous microspheres for efficient delivery of insulin and inhalation therapy of diabetes. Adv. Healthc. Mater..

[31] Chen B.Q., Kankala R.K., He G.Y., Yang D.Y., Li G.P., Wang P., Wang S.B., Zhang Y.S., Chen A.Z. (2018). Supercritical fluid-assisted fabrication of indocyanine green-encapsulated silk fibroin nanoparticles for dual-triggered cancer therapy. ACS Biomater. Sci. Eng..

[32] Xu P.Y., Kankala R.K., Pan Y.J., Yuan H., Wang S.B., Chen A.Z. (2018). Overcoming multidrug resistance through inhalable sirna nanoparticles-decorated porous microparticles based on supercritical fluid technology. Int. J. Nanomed..

[33] Prosapio V., De Marco I., Reverchon E. (2016). Pvp/corticosteroid microspheres produced by supercritical antisolvent coprecipitation. Chem. Eng. J..

[34] Padrela L., Rodrigues M.A., Duarte A., Dias A.M.A., Braga M.E.M., de Sousa H.C. (2018). Supercritical carbon dioxide-based technologies for the production of drug nanoparticles/nanocrystals—A comprehensive review. Adv. Drug Deliv. Rev..

[35] Reverchon E., De Marco I. (2011). Mechanisms controlling supercritical antisolvent precipitate morphology. Chem. Eng. J..

[36] Abuzar S.M., Hyun S.-M., Kim J.-H., Park H.J., Kim M.-S., Park J.-S., Hwang S.-J. (2018). Enhancing the solubility and bioavailability of poorly water-soluble drugs using supercritical antisolvent (sas) process. Int. J. Pharm..

[37] Puglia C., Lauro M.R., Tirendi G.G., Fassari G.E., Carbone C., Bonina F., Puglisi G. (2017). Modern drug delivery strategies applied to natural active compounds. Expert Opin. Drug Deliv..

[38] Archana D., Singh B.K., Dutta J., Dutta P.K. (2015). Chitosan-pvp-nano silver oxide wound dressing: In vitro and in vivo evaluation. Int. J. Biol. Macromol..

[39] Teodorescu M., Bercea M., Morariu S. (2019). Biomaterials of pva and pvp in medical and pharmaceutical applications: Perspectives and challenges. Biotechnol. Adv..

[40] Matos R.L., Lu T., Prosapio V., McConville C., Leeke G., Ingram A. (2019). Coprecipitation of curcumin/pvp with enhanced dissolution properties by the supercritical antisolvent process. J. CO_2_ Util..

[41] Prosapio V., De Marco I., Scognamiglio M., Reverchon E. (2015). Folic acid–pvp nanostructured composite microparticles by supercritical antisolvent precipitation. Chem. Eng. J..

[42] Wang B., Wang D.D., Zhao S., Huang X.B., Zhang J.B., Lv Y., Liu X.C., Lv G.J., Ma X.J. (2017). Evaluate the ability of pvp to inhibit crystallization of amorphous solid dispersions by density functional theory and experimental verify. Eur. J. Pharm. Sci..

[43] Bolten D., Türk M. (2011). Experimental study on the surface tension, density, and viscosity of aqueous poly(vinylpyrrolidone) solutions. J. Chem. Eng. Data.

[44] Galia A., Scialdone O., Filardo G., Spano T. (2009). A one-pot method to enhance dissolution rate of low solubility drug molecules using dispersion polymerization in supercritical carbon dioxide. Int. J. Pharm..

[45] Szafraniec J., Antosik A., Knapik-Kowalczuk J., Kurek M., Syrek K., Chmiel K., Paluch M., Jachowicz R. (2017). Planetary ball milling and supercritical fluid technology as a way to enhance dissolution of bicalutamide. Int. J. Pharm..

[46] Gokhale A., Khusid B., Dave R.N., Pfeffer R. (2007). Effect of solvent strength and operating pressure on the formation of submicrometer polymer particles in supercritical microjets. J. Supercrit. Fluids.

[47] De Marco I., Rossmann M., Prosapio V., Reverchon E., Braeuer A. (2015). Control of particle size, at micrometric and nanometric range, using supercritical antisolvent precipitation from solvent mixtures: Application to pvp. Chem. Eng. J..

[48] Tabasum S., Singh R.P. (2019). Fisetin suppresses migration, invasion and stem-cell-like phenotype of human non-small cell lung carcinoma cells via attenuation of epithelial to mesenchymal transition. Chem. -Biol. Interact..

